# Low-Temperature Ozone Sensors Based on Yb-Doped Urchin-like Hierarchical In_2_O_3_ Microspheres

**DOI:** 10.3390/nano16120745

**Published:** 2026-06-14

**Authors:** Xiumei Xu, Yi Zhou, Haijiao Zhang, Bao Wan, Yuhan Xu, Mengmeng Dai, Gui Wang, Gang Yang, Yongsheng Zhu

**Affiliations:** 1 College of Materials and New Energy, Nanyang Normal University, 1638 Wolong Road, Nanyang 473061, China; 2 College of Physics and Electronic Engineering, Nanyang Normal University, 1638 Wolong Road, Nanyang 473061, China

**Keywords:** In_2_O_3_, Yb-doped, urchin-like hierarchical structure, ozone sensing, low-temperature detection

## Abstract

As a highly oxidizing and toxic gas, ozone (O_3_) poses significant hazards to human health and the environment even at low concentrations. Therefore, the development of ozone gas sensors that can operate stably at low temperatures while simultaneously exhibiting high response, fast response characteristics, excellent selectivity, and long-term stability remains a crucial challenge in the field of gas sensing. In this work, Pure In_2_O_3_ and Yb-doped urchin-like hierarchical In_2_O_3_ microspheres were successfully synthesized via a one-step hydrothermal method. The crystal structure, morphological features, elemental composition, and band structure of the as-prepared samples were systematically characterized by XRD, FESEM, TEM, HRTEM, XPS, and UV–vis spectroscopy. Gas-sensing tests demonstrated that Yb doping significantly enhanced the ozone-sensing performance of In_2_O_3_. Among all the samples, the 3%Yb-doped In_2_O_3_ sensor exhibited the best response toward 1 ppm ozone at 40 °C, reaching approximately 1015, which was about 11 times higher than that of pristine In_2_O_3_. Meanwhile, the sensor showed a response time of 172 s. In addition, the 3%Yb-doped In_2_O_3_ sensor exhibited good repeatability, excellent selectivity, and long-term stability. The excellent gas-sensing performance can be attributed to the electronic structure modulation and increased O_V_-related oxygen defect component induced by Yb doping, as well as the enhanced gas diffusion and interfacial reaction capability provided by the urchin-like hierarchical structure.

## 1. Introduction

Ozone (O_3_) is a highly reactive and strongly oxidative gas that is widely used in disinfection and sterilization. However, excessive exposure to ozone in the environment can cause damage to the human respiratory system. Therefore, highly sensitive and real-time monitoring of ozone is of great significance [1,2,3,4]. Metal oxide semiconductor (MOS) gas sensors are considered promising candidates for ozone detection because of their low cost, fast response, and ease of integration. Among them, indium oxide (In_2_O_3_), as a typical n-type semiconductor, has attracted extensive attention in ozone sensing due to its high electron mobility and strong interaction with oxidizing gases [5,6]. Although several In_2_O_3_-based ozone sensors have been reported to operate at low or even room temperature, simultaneously achieving high sensitivity, excellent selectivity, and long-term stability under near-room-temperature conditions remains a significant challenge. This is mainly attributed to the insufficient utilization of active surface sites and the relatively sluggish surface reaction kinetics at low temperatures [7,8]. Similar issues are also observed in NO_2_- and CO_2_-sensing systems. Recent studies show that NO_2_-sensing performance can be improved via morphology control and surface modification, while functionalized metal oxides have also demonstrated enhanced CO_2_ detection under near-room-temperature conditions [9,10,11], indicating that surface and structural engineering are key to low-temperature gas sensing.

To improve the gas-sensing performance of In_2_O_3_, various strategies have been developed, including noble metal modification and element doping [12,13]. In particular, rare-earth dopants are effective in regulating electronic structure and surface oxygen species due to their unique 4f orbitals [14]. Among them, Yb^3+^, with an ionic radius close to In^3+^, can induce lattice modulation and oxygen vacancy formation while maintaining structural stability [15,16,17]. In addition, hierarchical nanostructures assembled from one-dimensional nanorods provide high surface area and open diffusion channels, which are beneficial for gas adsorption and reaction [18,19,20]. Therefore, combining rare-earth doping with hierarchical structure design is an effective strategy for improving near-room-temperature ozone sensing.

In this work, a Yb-doped nanorod-assembled urchin-like hierarchical In_2_O_3_ microstructure was successfully constructed via a hydrothermal method. By adjusting the doping concentration and growth conditions, a hierarchical structure composed of radially aligned nanorods was obtained, and its ozone-sensing performance at low temperatures was systematically investigated. The experimental results demonstrate that the material exhibits excellent ozone response behavior at low temperatures, along with good repeatability and stability. This study provides an effective strategy for realizing efficient ozone detection based on In_2_O_3_ at low temperatures and highlights the importance of the synergistic regulation of rare-earth doping and hierarchical structures in low-temperature gas sensing.

## 2. Experimental Section

### 2.1. Chemical Materials

Indium chloride tetrahydrate (InCl_3_·4H_2_O, 99.9%), Ytterbium(III) chloride hexahydrate (YbCl_3_·6H_2_O, 99.9%) and urea (99.5%) were obtained from Shanghai Aladdin Biochemical Technology Co., Ltd., Shanghai, China. In addition, absolute ethanol and deionized water were used for all of the experiments. All reagents were analytical grade and used as purchased without further purification.

### 2.2. Preparation of Pure In_2_O_3_ and Yb-Doped Urchin-like Hierarchical In_2_O_3_ Microspheres

Figure S1 illustrates the schematic diagram of the synthesis process. In a typical synthesis, 0.586 g (2 mmol) of indium(III) chloride tetrahydrate (InCl_3_·4H_2_O), a certain amount of ytterbium(III) chloride hexahydrate (YbCl_3_·6H_2_O, with doping concentrations of 0.5, 1, 3, and 5 mol%), and 0.6 g of urea were dissolved in 72 mL of deionized water, followed by magnetic stirring for 60 min to form a homogeneous solution. The resulting transparent solution was then transferred into a Teflon-lined stainless-steel autoclave, sealed, and maintained at 160 °C for 12 h. After natural cooling to room temperature, the obtained white precipitates were collected by centrifugation and washed several times with deionized water and ethanol. The samples with different Yb doping concentrations (0.5, 1, 3, and 5 mol%) were subsequently calcined at 550 °C for 4 h, and the resulting products were denoted as 0.5%Yb, 1%Yb, 3%Yb, and 5%Yb, respectively. For comparison, the undoped sample prepared under identical conditions without the addition of YbCl_3_·6H_2_O was denoted as Pure.

### 2.3. Material Characterization

XRD patterns were analyzed by an X-ray diffractometer (Rigaku Corporation, Tokyo, Japan) equipped with Cu Kα radiation (λ = 1.5406 Å). X-ray photoelectron spectroscopy (XPS) measurements were performed by a Thermo Scientific K-Alpha (Thermo Fisher Scientific, Waltham, MA, USA). The specific surface area and pore distribution of the samples were measured by Micromeritics ASAP 2460 (Micromeritics Instrument Corporation, Norcross, GA, USA). The nanoscale morphology of the sample was observed using a field-emission scanning electron microscope (SEM) model JSM-7800F (JEOL Ltd., Tokyo, Japan) and a transmission electron microscope (TEM) model JEM-F200 (JEOL Ltd., Tokyo, Japan). The absorption spectra were measured using a UV–Vis spectrophotometer (Shimadzu UV-2600, Shimadzu Corporation, Kyoto, Japan).

### 2.4. Fabrication and Measurement of Gas Sensor

**Figure 1:**
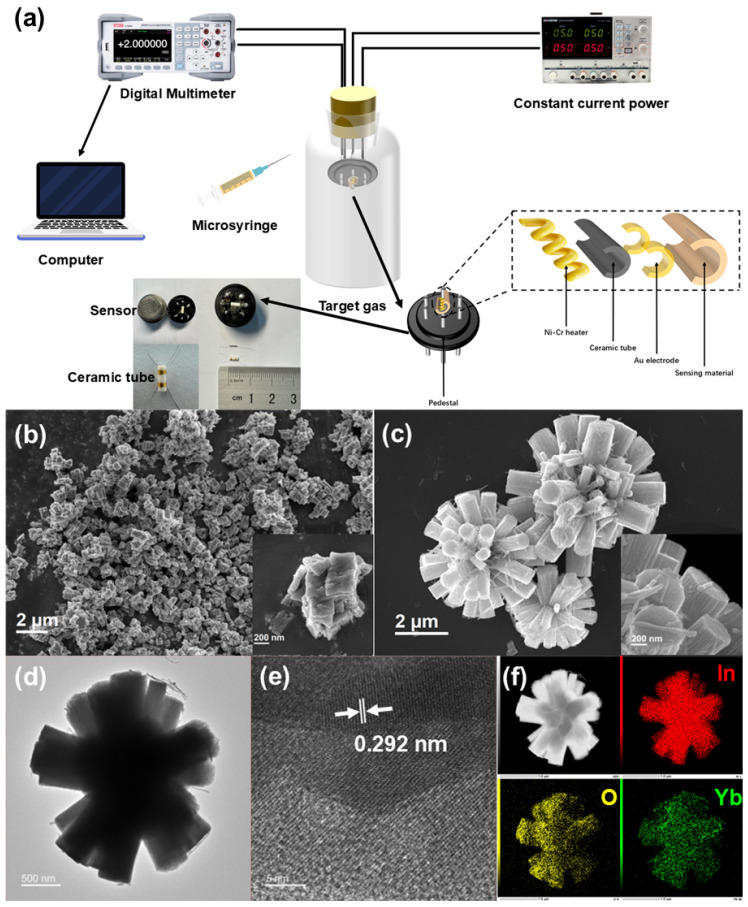
(**a**) Schematic of the sensors and the gas-sensing test equipment, SEM images of Pure (**b**) and 3%Yb (**c**), (**d**) TEM image of 3%Yb, (**e**) high-resolution TEM (HRTEM) image of 3%Yb, (**f**) elemental mapping of 3%Yb by EDS.

The gas sensor was fabricated by a conventional drop-coating method. Specifically, 10 mg of the as-prepared sample was dispersed in 2 mL of ethanol and thoroughly ground to obtain a homogeneous slurry. The slurry was then coated onto the outer surface of a commercial ceramic tube equipped with a pair of Pt lead wires and two Au electrodes. The operating temperature of the sensor was controlled by a Ni–Cr alloy heating wire placed inside the ceramic tube. After coating, the ceramic tube was calcined in a muffle furnace at 200 °C for 2 h to ensure good adhesion and electrical contact between the sensing layer and the ceramic substrate. The sintered ceramic tube was then welded to the sensor base together with the Ni–Cr heating wire and aged in air at 400 °C for 24 h to further improve the long-term stability and repeatability of the sensor. Ozone was generated using an ozone generator (FL-8A, Feili, Shenzhen, China). The ozone concentration in the bag was calibrated using an O_3_ calibrator (Model 306, 2B Technologies, Boulder, CO, USA). Gas-sensing performance was evaluated using a static testing system. The testing chamber was a glass vessel with a volume of approximately 1 L. The sensor was first placed in the chamber filled with fresh air until its resistance reached a stable baseline. A certain amount of target gas was then injected into the chamber using a microsyringe. After the sensor response reached a stable value, the sensor was transferred to another chamber filled with fresh air for recovery. During the recovery process, a brief thermal excitation treatment at 100 °C was applied to accelerate gas desorption and facilitate resistance recovery. The sensor was subsequently cooled back to the target operating temperature before the next measurement cycle [21]. The ambient temperature during the tests was approximately 25 °C, and the relative humidity ranged from 30% to 40%. The sensor response was defined as the resistance ratio, expressed as R_a_/R_g_ in reducing gases and R_g_/R_a_ in oxidizing gases, where R_g_ and R_a_ represent the resistance in the target gas and in air, respectively. The response/recovery times were defined as the time required for the sensor resistance to reach 90% of the total resistance change during the adsorption and desorption processes. Resistance signals were recorded using a UT8806 measurement system (UNI-TREND Technology Co., Ltd., Dongguan, China). The testing procedure and schematic illustration of the sensor configuration are shown in Figure 1a.

## 3. Results and Discussion

### 3.1. Structure and Morphology Characterization

The morphologies of the samples were examined by scanning electron microscopy (SEM) and transmission electron microscopy (TEM). As shown in Figure 1b–d and Figure S2a–g, the sample morphology varied significantly with increasing Yb doping concentration. At low doping levels, the incorporation of Yb into the In_2_O_3_ lattice induced moderate lattice distortion, which facilitated crystal nucleation. However, because the anisotropic growth effect was not yet prominent, the samples mainly exhibited dispersed nanorod-like structures. As the Yb doping concentration increased further, the dopant-induced lattice distortion gradually intensified, altering the relative growth rates of different crystal facets and favoring the oriented growth of nanorods along specific directions followed by radial self-assembly, leading to the formation of uniformly shaped urchin-like hierarchical structures [15,22]. However, when the Yb concentration was increased beyond this point, excessive lattice distortion compromised crystal structural stability. Meanwhile, an excessively high nucleation rate and kinetic imbalance during growth made it difficult for the nanounits to maintain an ordered arrangement [23,24,25]. Under the combined influence of these factors, the original urchin-like structures gradually collapsed. High-resolution transmission electron microscopy (HRTEM) images of the samples are shown in Figure 1e and Figure S2h. For 3%Yb and Pure, lattice spacings of 0.292 nm and 0.179 nm were observed, corresponding to the (222) and (440) planes of cubic In_2_O_3_ (c-In_2_O_3_), respectively. These results indicate that both 3%Yb and Pure belong to the c-In_2_O_3_ phase. In addition, the elemental mapping results of the 3%Yb and Pure (Figure 1f and Figure S2i) show that In and O elements are uniformly distributed in both samples, whereas Yb is uniformly distributed in the 3%Yb sample but absent in Pure. Furthermore, the TEM-EDS energy spectrum of the 3%Yb sample (Figure S3) exhibits the characteristic signal of Yb, further confirming the successful introduction of Yb into In_2_O_3_.

**Figure 2:**
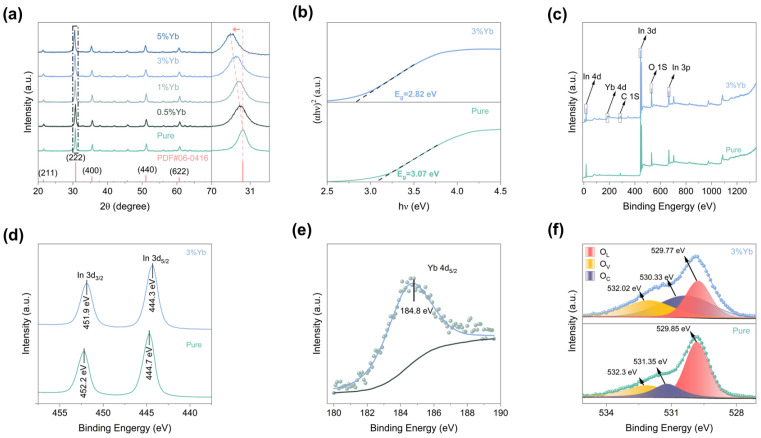
(**a**) XRD patterns of the samples. (**b**) UV–Vis diffuse reflectance spectra. (**c**) XPS survey spectra of Pure and 3%Yb. XPS spectra of (**d**) In 3d, (**e**) Yb 4d, (**f**) O 1s.

The XRD patterns of Pure and Yb-doped In_2_O_3_ (0.5–5%) samples are shown in Figure 2a. The diffraction peaks of the Pure sample can be assigned to cubic In_2_O_3_ (JCPDS No. 06–0416) [11]. For the Yb-doped samples, only the characteristic peaks of In_2_O_3_ are observed, with no diffraction peaks related to other Yb-containing compounds, indicating that no separate Yb phase was formed. Enlarged views of the (222) plane for the Pure and Yb-doped samples reveal that the diffraction peaks gradually shift slightly toward lower angles with increasing Yb content. This phenomenon indicates an increase in interplanar spacing and lattice expansion. This change may be attributed to the substitution of Yb^3+^ (0.87 Å) for In^3+^ (0.81 Å) in the In_2_O_3_ lattice, thereby inducing lattice distortion [24,26]. The regulation of the electronic structure provides a basis for the improvement of gas-sensing performance. The band gap energy (*E_g_*) of the samples can be calculated using Equation (1), and the results are shown in Figure 2b.

(1) $${\left(\alpha h\nu \right)}^{1/n}=A(h\nu -E_{g})$$

where *α*, *h*, *ν*, *E_g_*, and *A* represent the absorption coefficient, Planck’s constant, photon frequency, band gap energy, and a constant, respectively [27]. The calculated results indicate that the band gap of In_2_O_3_ decreases from 3.07 eV to 2.82 eV after Yb doping. This band gap narrowing facilitates electron migration and transfer during the gas-sensing process, thereby facilitating charge transfer during gas sensing. In addition, a narrower band gap can improve carrier mobility and contribute to enhanced thermal stability of the material, which further improves the overall operational stability of the device [27,28,29]. The elemental composition and chemical states of the Pure and 3%Yb were investigated by X-ray photoelectron spectroscopy (XPS). The survey spectra (Figure 2c) confirm that only In, O, and Yb elements are present in both samples, consistent with the EDS results. Due to the low doping concentration, the Yb-related signal intensity is relatively weak. The high-resolution In 3d spectra (Figure 2d) show a positive shift in binding energy after Yb doping, indicating a change in the chemical environment around In^3+^ ions [30]. Figure 2e presents the high-resolution Yb 4d spectrum of the 3%Yb sample, where the characteristic peak at 184.8 eV is attributed to Yb 4d_5/2_ [31]. The O 1s spectra of the Pure and 3%Yb samples were deconvoluted and are shown in Figure 2f. For the Pure sample, the peaks are located at 529.85, 531.35, and 532.3 eV, corresponding to lattice oxygen (O_L_), oxygen-deficient related oxygen species (O_V_), and chemisorbed oxygen (O_C_), respectively. As shown in Figure S4, the corresponding area percentages are 54%, 20%, and 26%, respectively. After Yb doping, the relative proportion of the O_V_ component increases significantly from 20% to 38%, while chemisorbed oxygen increases from 26% to 30%. The increase in O_V_ component plays a crucial role in enhancing the gas-sensing performance, as oxygen-deficient surface states serve as active sites for gas adsorption and subsequent surface reactions [29,32]. As shown in Figure S5, the N_2_ adsorption–desorption isotherms of the 3%Yb and Pure exhibit type IV characteristics with H3 hysteresis loops, indicating a mesoporous structure. Compared with the Pure sample, the 3%Yb shows a higher specific surface area. The increased surface area provides more active sites for gas adsorption, thereby enhancing gas uptake capacity and improving sensing performance [27].

### 3.2. Sensing Properties

**Figure 3:**
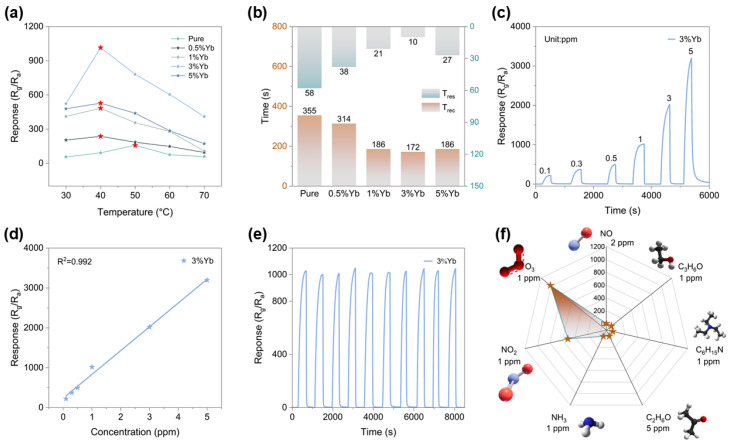
Gas-sensing performance of the sensors with a sensing temperature of 40 °C unless otherwise specified. (**a**) Response vs. operating temperature curves with different sensors toward 1 ppm of O_3_; (**b**) Response/recovery time of sensor toward 1 ppm O_3_. (**c**) Dynamic response curves of the 3%Yb sensor to various O_3_ concentrations. (**d**) Linear fitting between sensor responses and O_3_ concentrations for the 3%Yb sensor. (**e**) Repeatability of the 3%Yb sensor toward 1 ppm O_3_. (**f**) Responses of the 3%Yb sensor to various kinds of detected gases.

Figure 3a shows the effect of operating temperature on the sensing performance of Pure In_2_O_3_ and Yb-doped In_2_O_3_ (0.5–5 at%) toward 1 ppm O_3_. The Pure In_2_O_3_ sensor exhibits the highest response at 50 °C, whereas the optimum operating temperature of the Yb-doped sensors decreases to 40 °C. The response values of Pure, 0.5%Yb, 1%Yb, 3%Yb, and 5%Yb toward 1 ppm O_3_ are 91.73, 236.6, 482.38, 1015.28, and 527.93, respectively. These results indicate that Yb doping has a significant influence on the ozone response of the sensors, among which the 3%Yb shows the best sensing performance. In addition, the response and recovery times of the sensors toward 1 ppm O_3_ at their respective optimal operating temperatures were further analyzed. Figure 3b and Figure S6 present the dynamic response/recovery curves and the corresponding data under thermally assisted recovery conditions (brief heating to 100 °C). Clearly, the samples with different Yb doping levels exhibit obvious differences in response and recovery times. Among them, the 3%Yb shows the shortest response/recovery times of 172 s/10 s, which are reduced by 183 s/48 s, 142 s/28 s, 14 s/11 s, and 14 s/17 s compared with Pure, 0.5%Yb, 1%Yb, and 5%Yb, respectively. In addition, to evaluate the intrinsic recovery behavior at 40 °C without additional heating, the corresponding recovery curves were also measured (Figure S7).

Figure 3c,d and Figure S8 show the response variation in the sensing materials toward different O_3_ concentrations at 40 °C, together with the corresponding fitted curves. As the O_3_ concentration increases, the sensor response gradually increases, while good resistance stability is maintained during long-term continuous testing. The fitting correlation coefficients are 0.993 for Pure, 0.985 for 0.5%Yb, 0.983 for 1%Yb, 0.992 for 3%Yb, and 0.991 for 5%Yb. These results indicate that the O_3_ concentration in the environment can be accurately inferred from the variation in response values, indicating good quantitative detection capability. Figure 3e shows the repeatability of the 3%Yb sensor over 10 cycles under 1 ppm O_3_, demonstrating excellent short-term consistency. As shown in Figure S10, the sensor response fluctuates only within an acceptable range over 60 days without obvious degradation, indicating good long-term stability. Selectivity is an important parameter for gas sensors. As shown in Figure 3f, the 3%Yb exhibits a much stronger response to O_3_ than to other interfering gases, including NO_2_, NH_3_, ethanol, triethylamine, acetone, and NO. This excellent selectivity is mainly attributed to the distinct reaction pathways of O_3_ compared with other gases. As a strong oxidizing gas, O_3_ can directly capture electrons and form reactive oxygen species, enabling efficient charge transfer even at low temperatures. In contrast, NO_2_ shows relatively slower kinetics and its sensing process is more dependent on surface-adsorbed oxygen species, which are limited at low operating temperatures, resulting in a weaker response [33]. In addition, the responses to reducing gases are much lower due to the insufficient activation at 40 °C. Overall, the 3%Yb demonstrates good selectivity toward O_3_. Furthermore, the effect of relative humidity (RH) on the ozone-sensing performance of the 3%Yb sensor was investigated. Different humidity environments were established using ambient air (~32.6% RH), saturated K_2_CO_3_ solution (~43.2% RH), and saturated KCl solution (~84.3% RH). The dynamic response–recovery curves of the sensor toward 1 ppm O_3_ at 40 °C under different humidity conditions are shown in Figure S9. As the relative humidity increased, the sensor response gradually decreased. This behavior may be attributed to the adsorption of H_2_O molecules on the material surface and the formation of hydroxyl species through their dissociation, which occupy part of the active sites and compete with ozone-related surface reactions, thereby reducing the sensing response. Meanwhile, the 3%Yb sensor exhibits superior performance at low ozone concentrations (Table 1). For a more direct comparison with the In_2_O_3_ nanostructured films reported in Ref. [8], further performance tests were conducted. Under room-temperature conditions, the 3%Yb sensor exhibited a response of 21.57 to 0.08 ppm O_3_ and achieved complete recovery without thermal reset, demonstrating its superior gas-sensing performance.

### 3.3. Sensing Mechanism

**Figure 4:**
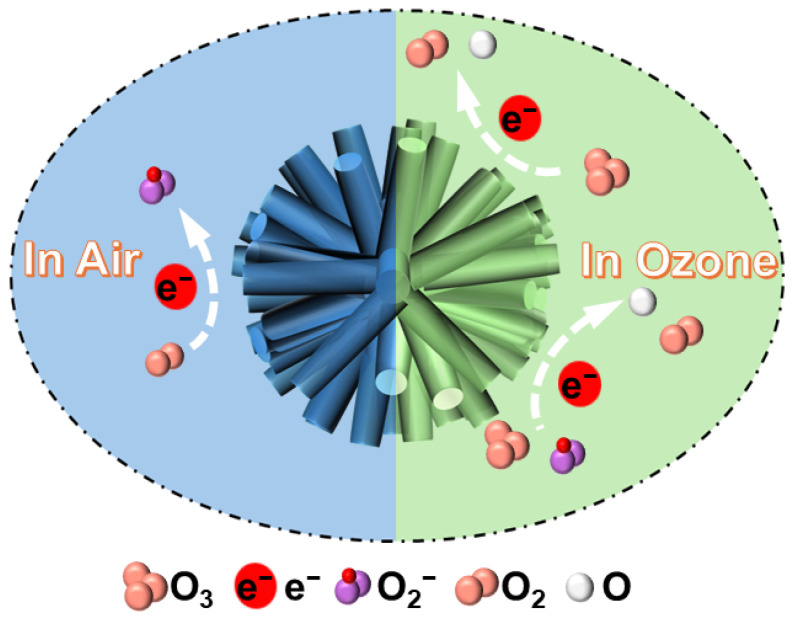
Schematic illustration of the gas-sensing mechanism of the 3%Yb sensor under air and O_3_ atmospheres.

The gas-sensing mechanism is illustrated in Figure 4. For surface resistive gas sensors based on n-type metal oxide semiconductors (MOS), the fundamental sensing process follows an “adsorption–reaction–desorption” pathway, in which the target gas interacts with the surface of the sensing material, leading to changes in electrical conductivity and thus variations in the electrical signal [7]. The electron transfer process results in corresponding changes in the material resistance. The dominant chemisorbed oxygen species on the surface depend on the operating temperature. At temperatures below 80 °C, O_2_^−^ is generally considered the dominant chemisorbed oxygen species on the surface [37,38,39]. The corresponding reactions are expressed in Equations (2)–(4):

(2) $$O_{2}+e^{-}\to O_{2}^{-}$$

(3) $$O_{3}+O_{2}^{-}+2e^{-}\to O_{2}+3O^{-}$$

(4) $$O_{3}+e^{-}\to O_{2}+O^{-}$$

In addition, the above discussion indicates that the enhanced gas-sensing performance is closely related to the high specific surface area provided by the hierarchical structure and the oxygen-deficient related surface states (O_V_) species. First, it should be noted that gas-sensing performance is strongly governed by morphological effects, which are fundamentally determined by surface reaction behavior. Hierarchically structured sensing materials generally exhibit higher utilization efficiency and therefore demonstrate superior gas-sensing properties compared with dense bulk materials [36]. In this work, the 3%Yb-doped sample exhibits an urchin-like hierarchical microsphere structure, which significantly improves the diffusion efficiency of gas molecules and surface reaction activity. According to the BET results, the specific surface area of the 3%Yb is also higher than that of the Pure In_2_O_3_ sample. Therefore, the improved sensing performance of the 3%Yb sensor can be mainly attributed to its larger specific surface area, which provides not only more gas diffusion pathways but also more active adsorption sites. Second, the O_V_ component is another factor affecting the gas-sensing process, and the increased O_V_ component promotes enhancement of the O_3_-sensing response. Compared with the Pure sample, the 3%Yb exhibits a significantly higher O_V_ component, which facilitates the ozone-sensing reaction. Third, the effective band gap of the 3%Yb is lower compared with that of the Pure sample, which facilitates electron excitation. Therefore, a more efficient electron transition process contributes to the excellent gas-sensing performance of the 3%Yb [29].

## 4. Conclusions

In this work, Yb-doped In_2_O_3_ urchin-like hierarchical structures assembled from nanorods were successfully synthesized via a hydrothermal method. For comparison, undoped In_2_O_3_ samples were prepared using the same procedure without the addition of the Yb source. Yb doping induces lattice distortion in In_2_O_3_, which further modulates its band structure and gas-sensing performance. Gas-sensing results demonstrate that Yb doping effectively enhances the ozone-sensing response of In_2_O_3_. Among all Yb-doped samples, the 3%Yb exhibits the highest response of approximately 1015 toward 1 ppm O_3_ at a relatively low operating temperature of 40 °C, together with a short response time of ~172 s. The improved performance can be attributed to several factors: (1) Yb-induced lattice distortion accompanied by band gap modulation, which optimizes the electronic structure and facilitates charge transport; (2) the increased O_V_ component introduced by Yb doping, providing more active sites for gas adsorption; and (3) the urchin-like hierarchical microsphere structure, which promotes gas diffusion and surface reaction kinetics.

This work provides a useful reference for the design of high-performance ozone-sensing materials and further deepens the understanding of the gas-sensing mechanism of Yb-doped In_2_O_3_ microspheres.

## Figures and Tables

**Table 1:** The comparison of the O_3_-sensing performance of metal oxide-based sensors between the reported literature and our work.

Samples	Concentration	Response	Condition	Ref.
In_2_O_3_ nanostructured films	0.08 ppm	2.2	RT	[8]
CuAlO_2_	1.15 ppm	≈1.9	250 °C	[34]
ZnCo_2_O_4_	0.89 ppm	23.3	200 °C	[35]
Mn_3_O_4_	5 ppm	≈0.2	RT	[36]
3%Yb	1 ppm	1015.28	40 °C	This work
3%Yb	0.08 ppm	21.57	RT	This work

## Data Availability

The original contributions presented in this study are included in the article/Supplementary Materials. Further inquiries can be directed to the corresponding authors.

## Supplementary Materials

The following supporting information can be downloaded at https://www.mdpi.com/article/10.3390/nano16120745/s1. Figure S1: Schematic of synthesis of urchin-like hierarchical In_2_O_3_ microspheres. Figure S2: SEM images of 0.5%Yb (a,b), 1%Yb (c,d) and 5%Yb (e,f); (g) TEM image of Pure; (h) high-resolution TEM (HRTEM) image of Pure; (i) elemental mapping of Pure by EDS. Figure S3: TEM-EDS energy spectra of 3%Yb. Figure S4: Oxygen species content. Figure S5: N2 adsorption–desorption isotherm of Pure and 3%Yb. Figure S6: Dynamic response curves of different sensors toward 1 ppm O_3_ at a sensing temperature of 40 °C: (a) Pure, (b) 0.5%Yb, (c) 1%Yb, (d) 3%Yb and (e) 5%Yb. Figure S7: Dynamic response curves of different sensors toward 1 ppm O_3_ at 40 °C: (a) Pure, (b) 0.5%Yb, (c) 1%Yb, (d) 3%Yb, and (e) 5%Yb. Figure S8: Dynamic response curves of the different sensors toward various O_3_ concentrations: (a) Pure, (b) 0.5%Yb, (c) 1%Yb, and (d) 5%Yb. Linear fitting plots of sensor responses versus O_3_ concentrations for (e) Pure, (f) 0.5%Yb, (g) 1%Yb, and (h) 5%Yb. Figure S9: (a–c) Dynamic response–recovery curves of the 3%Yb under different RH at 40 °C. Figure S10: Long-term stability of the 3%Yb sensor toward 1 ppm O_3_.
