# Supplementary material for: Low-Temperature Ozone Sensors Based on Yb-Doped Urchin-like Hierarchical In2O3 Microspheres
Source: Nanomaterials (Basel). 2026 Jun 14;16(12):745. doi: 10.3390/nano16120745 (PMC13306073; doi:10.3390/nano16120745)
Supplement: Supplementary file 1 [file nanomaterials-16-00745-s001.zip › nanomaterials-4353788-supplementary.pdf]

Supporting Information

Low-Temperature Ozone Sensors Based on Yb-Doped Urchin-like Hierarchical In_2O_3 Microspheres

Xiumei Xu [1], Yi Zhou [2], Haijiao Zhang [1,*], Bao Wan [2], Yuhan Xu [2], Mengmeng Dai [2], Gui Wang [2], Gang Yang [2] and Yongsheng Zhu [2,*]

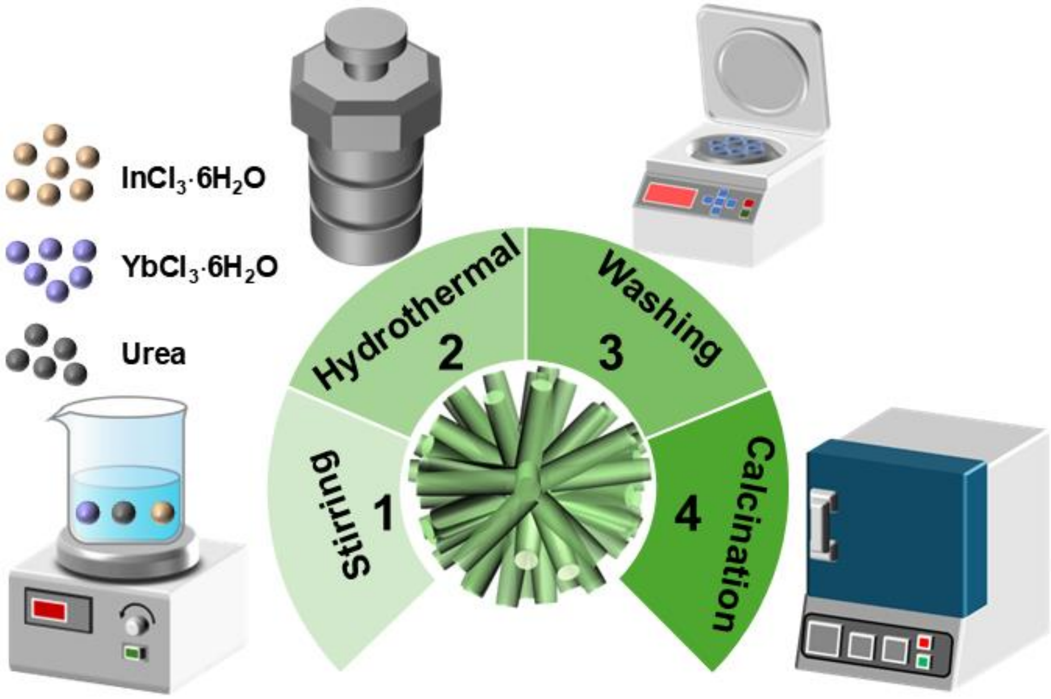

Figure S1: Schematic of synthesis of urchin-like hierarchical In_2O_3 microspheres.

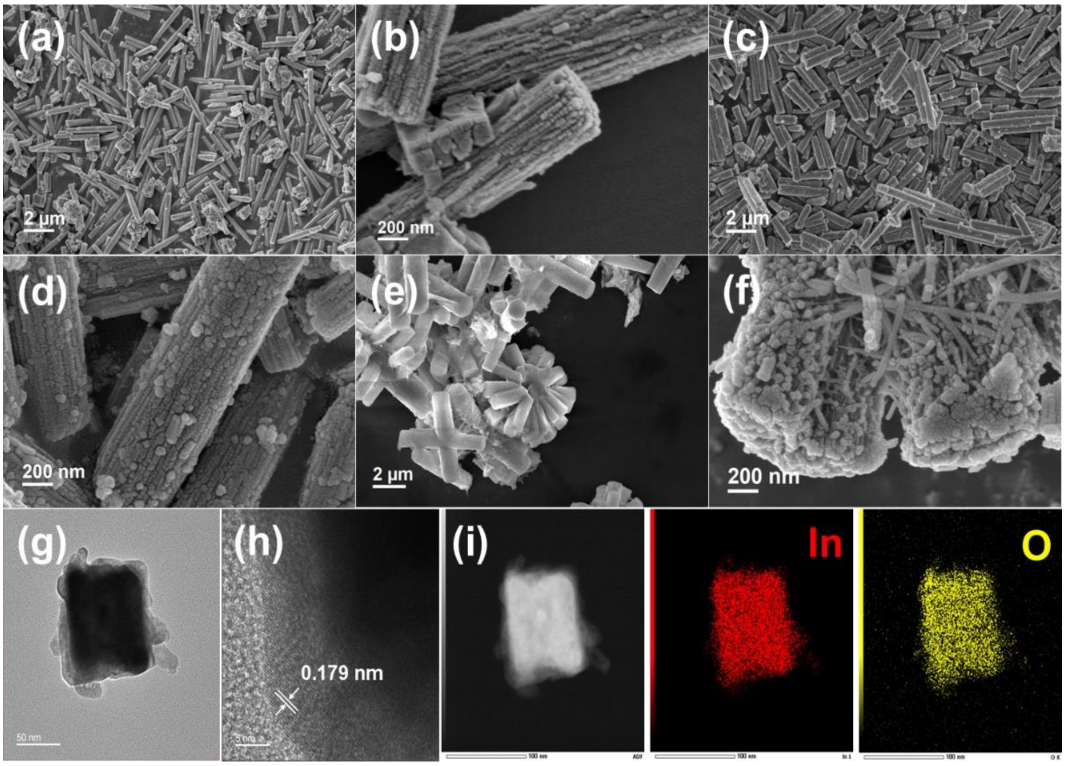

Figure S2: SEM images of 0.5%Yb (**a**,**b**), 1%Yb (**c**,**d**) and 5%Yb (**e**,**f**), (**g**) TEM image of Pure, (**h**) high-resolution TEM (HRTEM) image of Pure, and (**i**) elemental mapping of Pure by EDS.

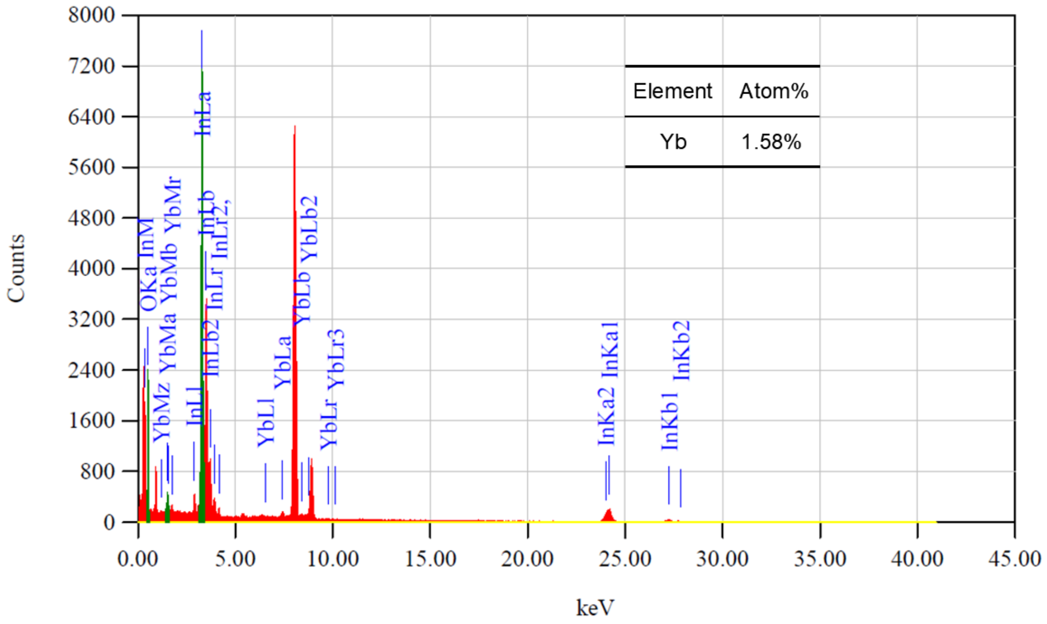

Figure S3: TEM-EDS energy spectra of 3%Yb.

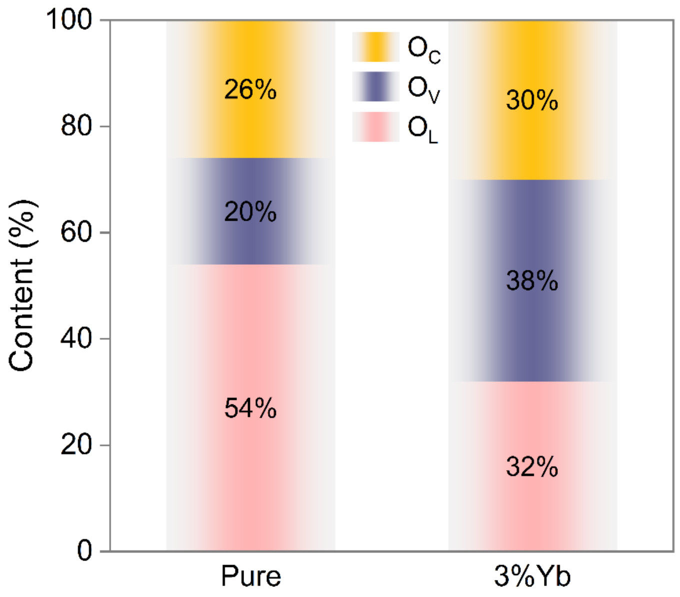

Figure S4: Oxygen species content.

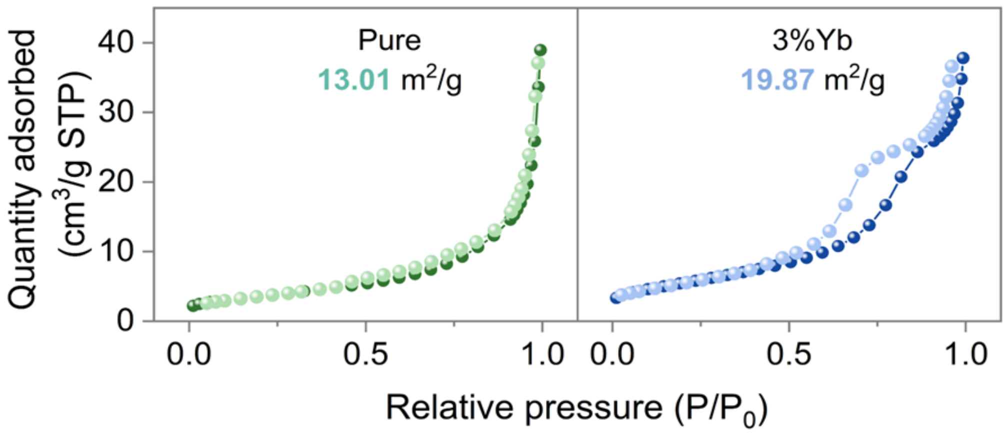

Figure S5: N_2 adsorption–desorption isotherm of Pure and 3%Yb.

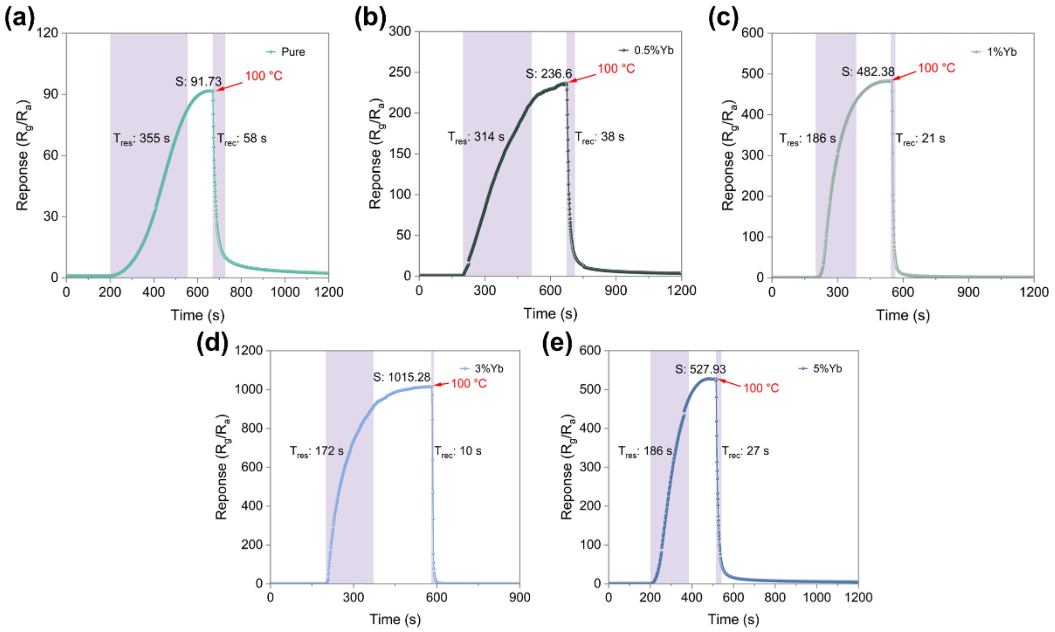

Figure S6: Dynamic response curves of different sensors toward 1 ppm O_3 at a sensing temperature of 40 °C: (**a**) Pure, (**b**) 0.5%Yb, (**c**) 1%Yb, (**d**) 3%Yb, and (**e**) 5%Yb.

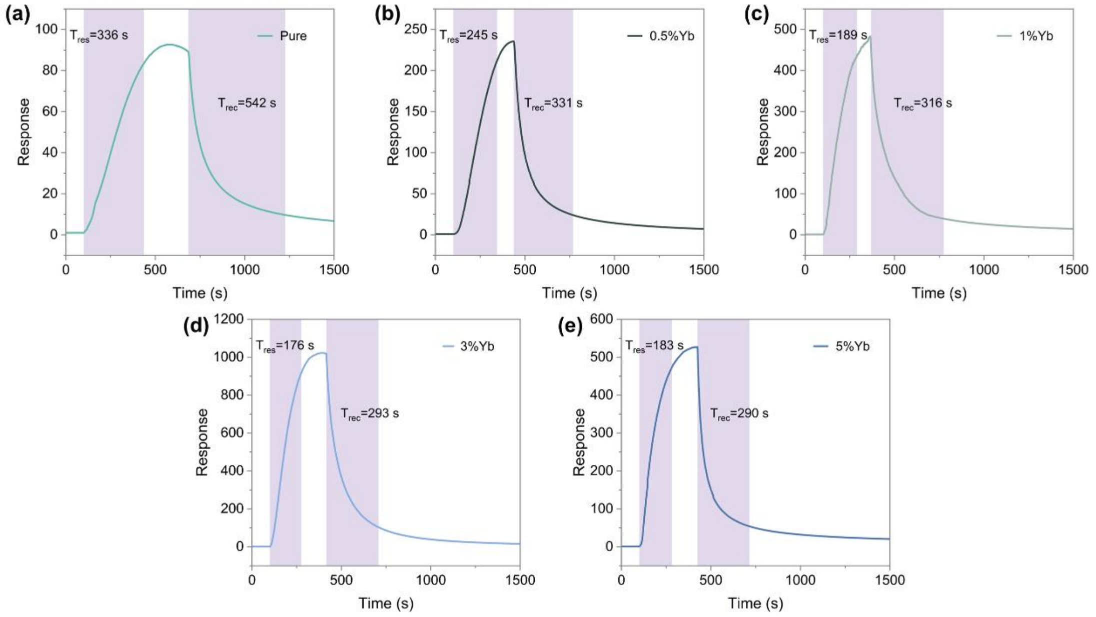

Figure S7: Dynamic response curves of different sensors toward 1 ppm O_3 at 40 °C: (**a**) Pure, (**b**) 0.5%Yb, (**c**) 1%Yb, (**d**) 3%Yb, and (**e**) 5%Yb.

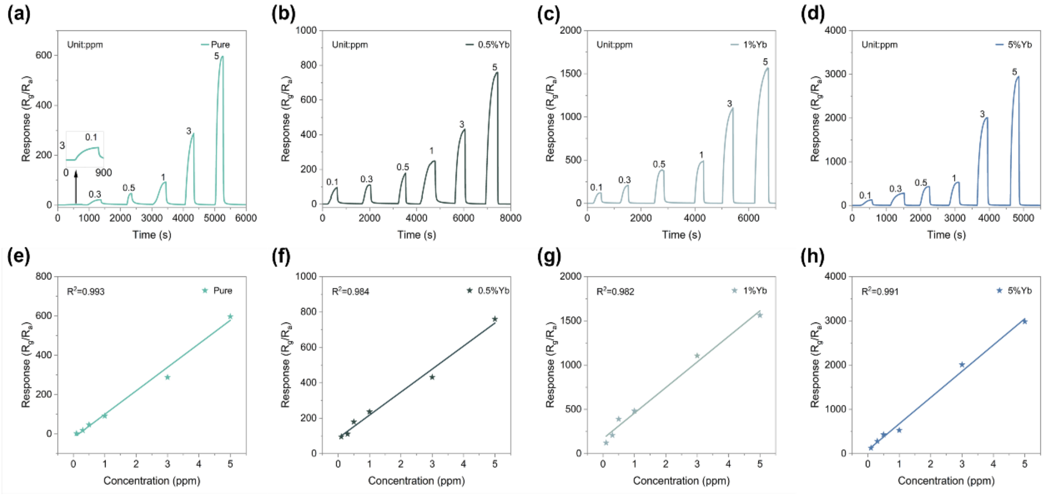

Figure S8 Dynamic response curves of the different sensors toward various O_3 concentrations: (**a**) Pure, (**b**) 0.5%Yb, (**c**) 1%Yb, and (**d**) 5%Yb. Linear fitting plots of sensor responses versus O_3 concentrations for (**e**) Pure, (**f**) 0.5%Yb, (**g**) 1%Yb, and (**h**) 5%Yb.

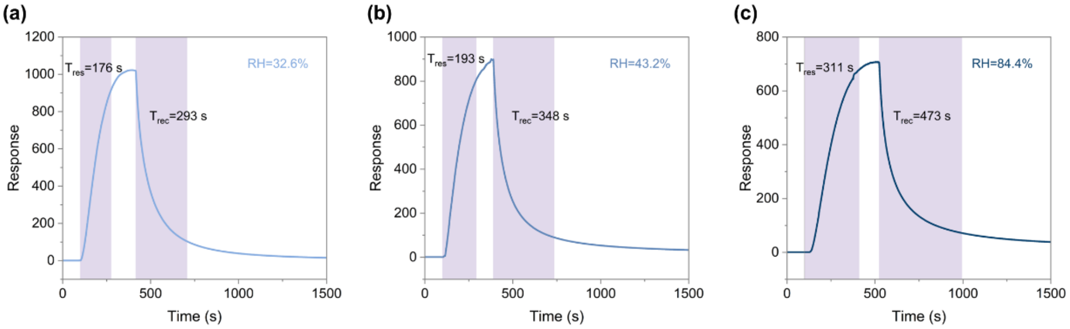

Figure S9: (a-c) Dynamic response–recovery curves of the 3%Yb under different RH at 40 °C.

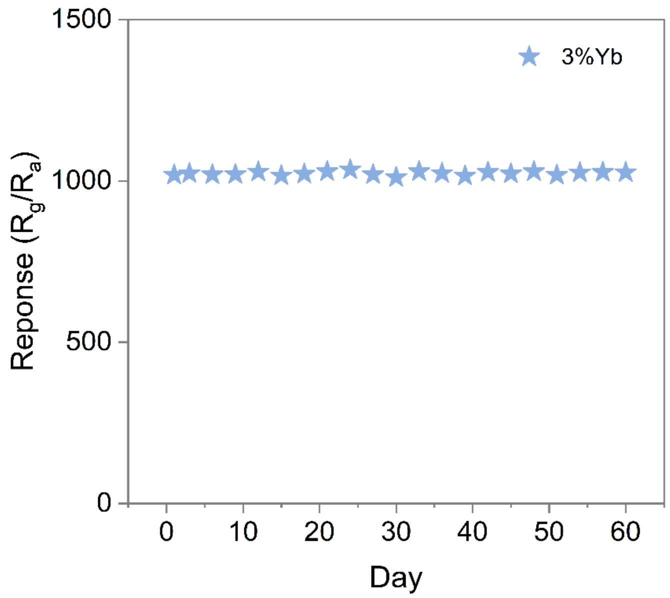

Figure S10: Long-term stability of the 3%Yb sensor toward 1 ppm O_3.
